# In Situ Synthesis of Magnetic Poly(DMAEAB-co-NIPAm)@Fe_3_O_4_ Composite Hydrogel for Removal of Dye from Water

**DOI:** 10.3390/gels7040201

**Published:** 2021-11-05

**Authors:** Zhi Chen, Xia Song, Wilson Wee Mia Soh, Yuting Wen, Jingling Zhu, Miao Zhang, Jun Li

**Affiliations:** 1 Department of Biomedical Engineering, National University of Singapore, 7 Engineering Drive 1, Singapore 117574, Singapore; z.chen@cqut.edu.cn (Z.C.); a0045788@u.nus.edu (X.S.); wilson.soh@u.nus.edu (W.W.M.S.); bieweny@nus.edu.sg (Y.W.); erizhuj@nus.edu.sg (J.Z.); zhangmiao@u.nus.edu (M.Z.); 2 School of Chemistry and Chemical Engineering, Chongqing University of Technology, Chongqing 400054, China

**Keywords:** magnetic hydrogel, composite, adsorption, bromophenol blue, water treatment

## Abstract

Water pollution by toxic substances, such as dye molecules, remains a major environmental problem that needs to be solved. In the present work, the magnetic composite hydrogel based on the poly(2-(methacryloyloxy)-*N*-(2-hydroxyethyl)-*N*,*N*-dimethylethan-1-aminium bromide-co-*N*-isopropylacrylamide) copolymer with incorporated Fe_3_O_4_ particles ((poly(DMAEAB-co-NIPAm)@Fe_3_O_4_)) was prepared by an in situ synthesis technique for the efficient removal of dye molecules from water. The successfully synthesized magnetic hydrogel was characterized by FTIR, XRD, TGA, and TEM. The removal efficiency of the anionic dye bromophenol blue (BPB) and the cationic dye rhodamine B (RDM) by the prepared hydrogel adsorbents was evaluated. Various adsorption parameters, including the concentration of adsorbents and adsorption time, were also investigated. The results showed that the synthesized magnetic hydrogel had excellent BPB removal performance compared to the removal of RDM. The optimum adsorbent concentration for 0.5 mM BPB solution was approximately 0.5 g/L, and the removal efficiency was more than 99%. The kinetics data of BPB removal fitted well into the pseudo-2nd-order model, indicating that BPB dye adsorption involves chemical adsorption and physical adsorption. In addition, recycling studies were conducted to examine the reusability of the magnetic hydrogel for BPB removal for up to five cycles and the hydrogel could be reused without losing its high removal efficiency. The magnetic hydrogel poly(DMAEAB-co-NIPAm)@Fe_3_O_4_ with high removal efficiency, good selectivity, and reusability shows great potential for the removal of anionic dyes in wastewater treatment.

## 1. Introduction

The dyestuff industry is important to the economy and many other industrial sectors, because a large number of synthetic dyes are applied in textile, leather, printing, paper, cosmetic, and food industries. Unfortunately, 10–15% of the dyes are eventually discharged into industrial effluents, becoming major environmental pollutants [1,2]. These dye molecules or their metabolites may be highly toxic, potentially carcinogenic, and even cause organic mutations or allergies in exposed organisms. They not only pollute the environment but also cross the entire food chain [3,4,5]. At present, the main treatment methods for dye wastewater are chemical oxidation, electro-coagulation, membrane filtration, biodegradation, flocculation, and adsorption [6,7,8,9,10,11]. Compared with other treatment methods, the adsorption method has become the most effective technique to remove dye or heavy metal ions from wastewater in the chemical, pharmaceutical, biological, and environmental industries because of its advantages of being eco-friendly, simple to operate, low energy consuming, and exhibiting great performance [12,13]. Therefore, it is an inevitable trend to develop highly selective and environmentally friendly adsorptions with high adsorption capacity.

Hydrogels, as one of the most important adsorbents, have been widely used as superabsorbent materials, water shutoff agents for petroleum recovery, filling materials for biological soft tissues, water-proof concrete additives, etc., in many fields such as health, chemical industry, biomedicine, and architecture [14,15,16]. There have been increasing studies to develop hydrogels for pollutant removal from water based on host–guest interactions. One example is a β-cyclodextrin-based hydrogel to remove organic pollutants from water [17]. At present, progress has also been made in the preparation of stimulus-responsive hydrogels, such as the thermosensitive hydrogel, the pH-responsive hydrogel, the magnetic responsive hydrogel, and the electric field responsive hydrogels [18,19,20,21,22,23,24,25,26,27]. Thermoresponsive hydrogels are among the most widely studied stimuli-responsive hydrogels. Poly(*n*-isopropylacrylamide) (PNIPAm) is an ideal thermoreponsive polymer because of its phase transition properties in response to temperature changes, and it has been used in functional materials for biological and biomedical applications and environmental sustainability [28,29,30,31,32,33,34,35]. A PNIPAm-based thermoresponsive smart adsorption system has been developed for efficient copper ion removal from water [18]. Magnetic hydrogels have also been developed for adsorption of contaminants from water because they could be easily separated from the solution by an external magnetic field [30]. Therefore, the molecular structure design of environmental-stimulus-responsive hydrogel is highly useful for improving the adsorption capacity and selectivity of the adsorbents by making use of specific interactions between the adsorbents and the adsorbates.

In this work, we synthesized and characterized a magnetic composite hydrogel based on poly(2-(methacryloyloxy)-*N*-(2-hydroxyethyl)-*N*,*N*-dimethylethan-1-aminium bromide-co-*N*-isopropylacrylamide) (poly(DMAEAB-co-NIPAM)) cationic copolymers with incorporated Fe_3_O_4_ particles using a simple in situ synthesis method. The magnetic (poly(DMAEAB-co-NIPAM)@Fe_3_O_4_) composite hydrogel was investigated for its effectiveness as an adsorbent in the removal of the anionic dye bromophenol blue (BPB) and the cationic dye rhodamine B (RDM). The effects of variables, such as adsorbent concentrations (g/L) and adsorption time (min), were studied and optimized. In addition, the adsorption process was further explored by fitting the data into adsorption kinetic models. The regeneration properties of the hydrogel were also investigated, demonstrating its potential reusability and simple fast separation characteristics in the adsorption and desorption of the dye molecules.

## 2. Experimental Section

### 2.1. Materials

*N*-isopropyl acrylamide (NIPAm, A.R. grade) was obtained from Tokyo Chemical Industry (TCI) Co., LTD., Tokyo, Japan. 2-Dimethylaminoethyl methacrylate (DMAEMA, A.R. grade), 2-bromoethanol (A.R. grade), *N*,*N*’-methylenebisacrylamide (MBA, C.P. grade), iron(III) chloride (FeCl_3_, reagent grade, ≥97%), sodium sulfite (Na_2_SO_3_, A.R. grade), potassium persulfate (KPS, ACS grade, ≥99%), bromophenol blue (BPB, electrophoresis reagent grade), rhodamine B (RDM, reagent grade, ≥95%), and ammonium hydroxide solution (ACS reagent, 28–30%, NH_3_ basis) were purchased from Sigma–Aldrich Chemical Company, Inc. (St. Louis, MO, USA) Other reagents were all analytical grade, and all solutions were prepared with deionized (DI) water (the electrical resistivity was 18 MΩ∙cm at 25 °C, Millipore Direct-Q^®^5UV water purification system).

### 2.2. Synthesis of Poly(DMAEAB-co-NIPAm) Hydrogel

The poly(DMAEAB-co-NIPAm) hydrogel was synthesized with a one-pot method by free radical copolymerization of DMAEMA and NIPAm in aqueous medium, using 2-bromoethanol as the cationic quaternary ammonium reagent, MBA as the crosslinker, and KPS as the initiator. In brief, DMAEMA (3.4659 g, 0.02 mol), NIPAm (2.4790 g, 0.02 mol) monomers, and 2-bromoethanol (2.7217 g, 0.02 mol) in 50 mL of water was stirred in a 150 mL three-neck round-bottom flask; MBA (0.1181 g, 2 wt% with respect to the weight of DMAEMA and NIPAm) was added into the reaction mixture and maintained at 50 °C for 4 h with nitrogen gas. Then, the KPS initiator (59.1 mg, 1 wt% based on weight percentages of DMAEMA and NIPAm) was dissolved in 10 mL of water and added into the reaction mixture under nitrogen gas. The polymerization reaction continued for 4 h at 50 °C until a colorless transparent poly(DMAEAB-co-NIPAm) hydrogel was obtained. Next, the obtained hydrogel was shattered and immersed in DI water for 3 days (water was changed every 24 h) to remove the unreacted or soluble materials. Finally, the hydrogel was freeze-dried to afford the poly(DMAEAB-co-NIPAm) hydrogel powder and designated as P100. A control hydrogel, P0, was synthesized following the same procedures for preparing P100, without the addition of 2-bromoethanol.

### 2.3. Synthesis of Poly(DMEMAB-co-NIPAm)@Fe_3_O_4_ Magnetic Hydrogel

The magnetic hydrogel was synthesized according to the protocol adapted from the literature [36]. Briefly, 0.25 g FeCl_3_ in 20 mL of DI water was mixed with 1 mL of 4.8 wt% sodium sulfite aqueous solution and stirred vigorously at room temperature for 1 h under nitrogen atmosphere to reach equilibrium. One gram of P100 hydrogel powder was added into the prepared iron cation solution and swelled for 24 h to absorb all the solution. Subsequently, 5 mL of ammonium hydroxide solution was added to and rinsed with the swollen hydrogel for 4 h. Then, the swollen hydrogel, which changed to a black color, was washed with water until the pH of the solution became neutral and dried at 70 °C in a vacuum oven to obtain the P100@Fe_3_O_4_ magnetic hydrogel. A control hydrogel, P0@Fe_3_O_4_, was prepared following the same procedures for preparing P100@Fe_3_O_4_, using P0 instead of P100.

### 2.4. Characterizations

Fourier transform infrared (FTIR) spectra of samples in potassium bromide (KBr) pellets were recorded on an Agilent Cary 600 spectrophotometer (Agilent Co., Ltd., Palo Alto, CA, USA) in the range of 400–4000 cm^−1^. Scanning electron microscope (SEM) and energy-dispersive X-ray spectroscopy (EDS) studies were carried out using a Regulus 8230 (HITACHI Co., Ltd., Tokyo, JPN) equipped with an Ultim Extreme (Oxford Instruments, Oxford, UK). Thermogravimetric analysis (TGA) thermograms were measured with a thermogravimetric analyzer (NETZSCH STA 2500, Selb, Bavaria, Germany). The scanning temperature range was from room temperature to 700 °C and the heating rate was 10 °C/min. The morphology and size of the particles were characterized by transmission electron microscopy (TEM, JEM-2010F, JEOL Co., Ltd., Tokyo, JPN). The samples were dispersed in absolute ethanol by ultrasonication and dripped on copper mesh and measured in the dry state with 200 kV. The crystalline structure of the samples was determined using X-ray diffraction (XRD, Bruker Single Crystal D8 Venture, Bruker Co., Ltd., Karlsruhe, Germany). The scan range (2*θ*) was from 20 to 65°, and the scanning step frequency was 1 (°)/min. The ultraviolet–visible (UV–Vis) measurements of the solutions were tested using a UV–Vis spectrophotometer (UV-2600, SHIMADZU Corp., Kyoto, Japan).

### 2.5. Dye Removal Experiments

**Figure 1:**
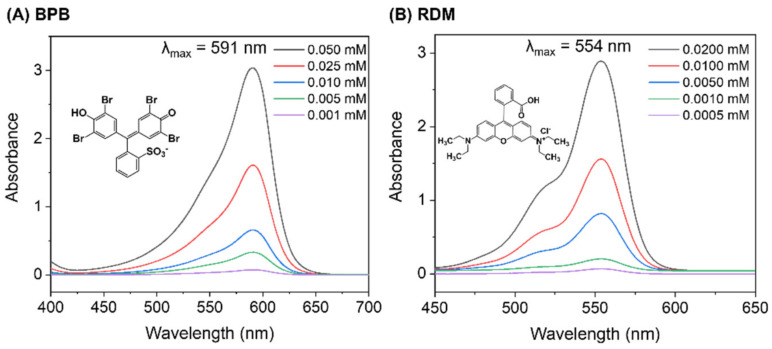
The chemical structures of BPB dye (**A**) and RDM dye (**B**) and their UV–Vis spectra at various concentrations.

In the batch adsorption studies to investigate the optimum adsorbent concentrations for BPB and RDM dye removal, different amounts (0–50 mg) of the non-magnetic or magnetic hydrogel adsorbents were added separately into 20 mL of the BPB dye solution (0.5 mM, 350 mg/L) or RDM dye solution (0.01 mM, 5 mg/L) in a glass vial and stirred for 120 min. In the kinetic studies of dye removal, 0.42 g/L of the adsorbent was used for BPB solution (initial concentration of 350 mg/L), and 0.62 g/L of the adsorbent was used for RDM solution (initial concentration of 5 mg/L) for a period of 2 h. After a predetermined interval of time, the P0@Fe_3_O_4_ and P100@Fe_3_O_4_ hydrogels were separated from the solution by an external magnet, while the P0 and P100 hydrogels were separated by centrifugation. The supernatant was taken to measure its absorbance to determine the dye concentration after adsorption. All experiments were conducted at 25 °C. A series of dye solutions with different concentrations were prepared to produce a standard curve by measuring the absorbance with a UV–Vis spectrophotometer from 400 to 700 nm at a scanning speed of 2 nm/s at room temperature. The chemical structures of the dyes and their UV–Vis spectra at various concentrations are shown in Figure 1. The wavelength of maximum absorbance, λ_max_, was 591 nm for BPB and 554 nm for RDM. The standard curve equations for the dyes were also generated: BPB dye, y = 60.564x + 0.0403 with *R*^2^ = 0.9991; RDM dye, y = 143.65x + 0.06 with *R*^2^ = 0.9978.

The residual concentration of the dye solution was determined using the standard curve. Equations (1) and (2) were used to calculate the equilibrium adsorption capacity (*q_e_*) and the removal efficiency (*E*) of dye, respectively.

(1) $$q_{e}=\frac{\left(C_{0}-C_{e}\right)V}{m}$$

(2) $$E=\frac{C_{0}-C_{e}}{C_{0}}\times 100\%$$

where *C*_0_ and *C_e_* (mg/L) are the initial and equilibrium concentration of the dye, respectively. *V* (L) is the solution volume, and *m* (g) is the mass of adsorbent used.

The kinetics data for dye adsorption were fitted into pseudo-1st-order and pseudo-2nd-order models with the following expressions (i.e., (3) and (4)), respectively:

(3) $$\ln\left(q_{e}-q_{t}\right)=\ln q_{e}-k_{1}t$$

(4) $$\frac{t}{q_{t}}=\frac{1}{k_{2}q_{e}^{2}}+\frac{t}{q_{e}}$$

where *q_e_* and *q_t_* are the dye adsorption capacity (mg g^−1^) at equilibrium and time *t*, respectively, and *k*_1_ (min^−1^) and *k*_2_ (g mg^−1^ min^−1^) are the pseudo-1st-order and pseudo-2nd-order rate constants, respectively.

### 2.6. Recycling Studies

After an adsorption experiment using 1 g/L of the adsorbents in 0.5 mM BPB solution, the desorption of BPB from the magnetic hydrogel P100@Fe_3_O_4_ and the non-magnetic hydrogel P100 was carried out in weakly acidic solutions (pH = 6) at 25 °C to regenerate the adsorbents before they were used again in subsequent cycles. The washing time needed to desorb BPB dye from the magnetic hydrogel P100@Fe_3_O_4_ and the non-magnetic hydrogel P100 was 2 and 5 min, respectively. The adsorption/desorption process was conducted five times.

## 3. Results and Discussion

### 3.1. Synthesis of Poly(DMAEAB-co-NIPAm)@Fe_3_O_4_ Magnetic Composite Hydrogel

**Figure 2:**
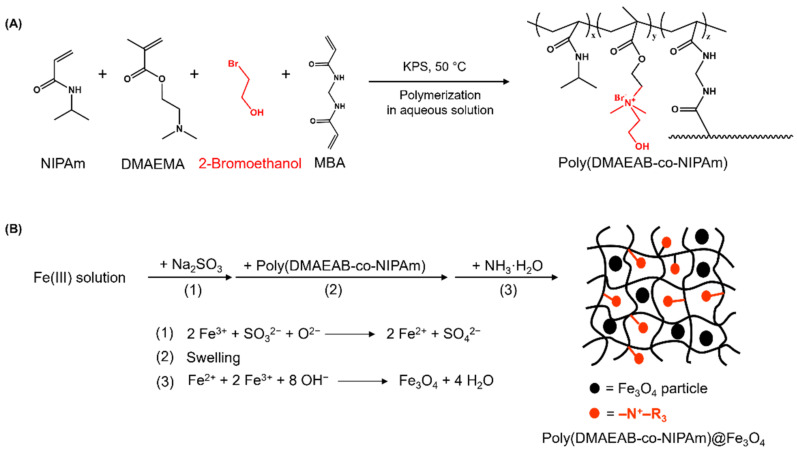
Synthesis scheme of (**A**) poly(DMAEAB-co-NIPAm) (P100) non-magnetic hydrogel and (**B**) poly(DMAEAB-co-NIPAm)@Fe_3_O_4_ (P100@Fe_3_O_4_) magnetic hydrogel.

The poly(DMAEAB-co-NIPAm) (P100) non-magnetic hydrogel and poly(DMAEAB-co-NIPAm)@Fe_3_O_4_ (P100@Fe_3_O_4_) magnetic hydrogel were successfully synthesized according to the protocol described in Figure 2. The control samples, poly(DMAEMA-co-NIPAm) (P0) non-magnetic hydrogel and poly(DMAEMA-co-NIPAm)@Fe_3_O_4_ (P0@Fe_3_O_4_) magnetic hydrogel, were also synthesized following the same protocol but without the addition of 2-bromoethanol. The synthesis parameters for the copolymer hydrogels are summarized in Table 1.

### 3.2. Characterizations of Poly(DMAEAB-co-NIPAm)@Fe_3_O_4_ Magnetic Composite Hydrogel

**Figure 3:**
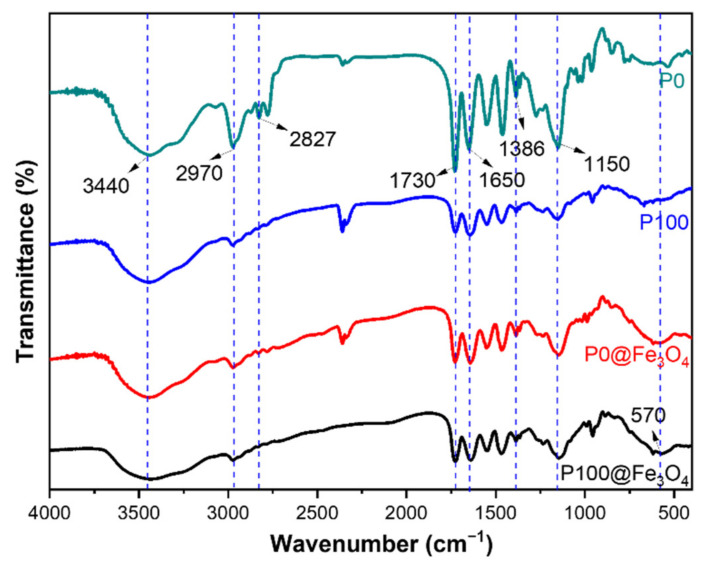
FTIR spectra of the non-magnetic hydrogels (i.e., P0 and P100) and the magnetic hydrogels (i.e., P0@Fe_3_O_4_ and P100@Fe_3_O_4_).

The FTIR spectra of the synthesized hydrogels are shown in Figure 3. The peaks around 1650 cm^−1^ and 1386 cm^−1^ and around 2970 cm^−1^ and 2827 cm^−1^ corresponded to the characteristic absorption peaks of C=O, –CH(CH_3_)_2_ and C–H of NIPAm of the P0 and P100 hydrogels, respectively. The peaks around 1730 cm^−1^ and 1150 cm^−1^ corresponded to the characteristic absorption peaks of C=O and C–O of DMAEMA of the hydrogel. After the introduction of Fe_3_O_4_ particles, the characteristic absorption peaks of each functional group were still present, but the intensity was weakened in the magnetic hydrogel P0@Fe_3_O_4_ and P100@Fe_3_O_4_, and the characteristic peaks of Fe–O were very obvious, near 570 cm^−1^. Therefore, the FTIR analysis showed that the non-magnetic hydrogels (i.e., P0 and P100) and the magnetic hydrogels (i.e., P0@Fe_3_O_4_ and P100@Fe_3_O_4_) were successfully prepared.

**Figure 4:**
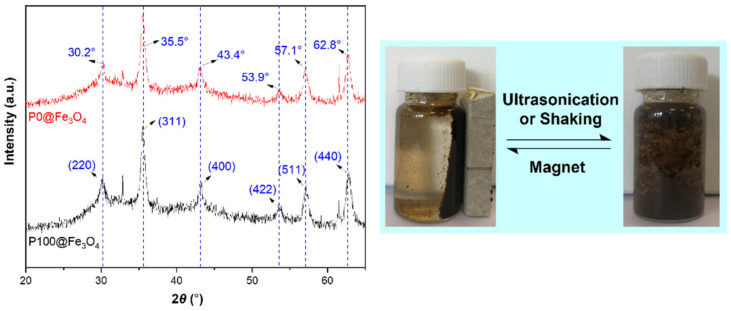
XRD diffractograms of the magnetic hydrogels (**left**) and photographs of P100@Fe_3_O_4_ particles in deionized water (**right**).

The XRD patterns of the magnetic hydrogels P0@ Fe_3_O_4_ and P100@ Fe_3_O_4_ were measured to analyze their crystalline structures and compositions (Figure 4 (left)). There were obvious diffraction peaks at 30.2, 35.5, 43.4, 53.9, 57.1, and 62.8° that responded to the (220), (311), (400), (422), (511), and (440) crystal planes of cubic phase Fe_3_O_4_, respectively [37]. These results indicate that the Fe_3_O_4_ particles were successfully introduced into the magnetic hydrogels P0@Fe_3_O_4_ and P100@Fe_3_O_4_, and the organic components had no effect on the crystallinity of Fe_3_O_4_. Moreover, the magnetic hydrogel particles had good dispersions in deionized water (Figure 4 (right)). When the magnet was close to the dispersions, obvious magnetic separation occurred quickly. P100@Fe_3_O_4_ particles were attracted to the wall of the vial near the magnetic field, which showed that the prepared Fe_3_O_4_ particles inside the hydrogel had good paramagnetism. After removing the magnet, the P100@Fe_3_O_4_ hydrogel particles re-dispersed into the solution under ultrasonication or shaking.

**Figure 5:**
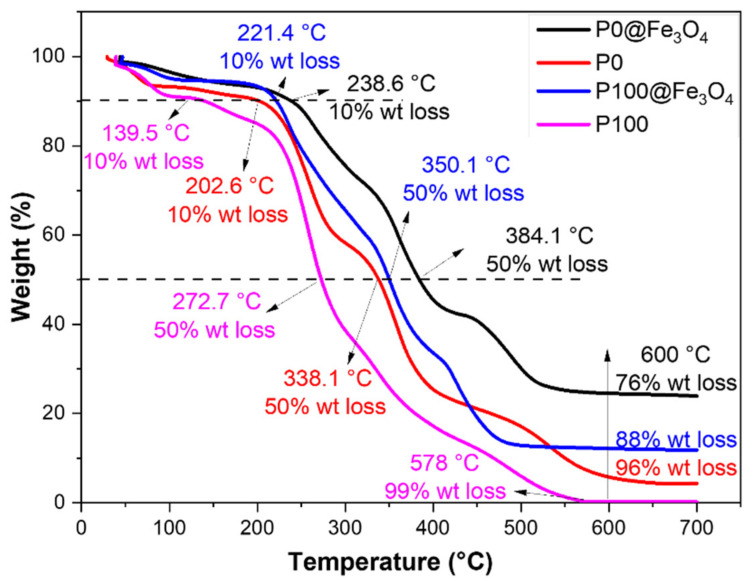
TGA thermograms of the non-magnetic hydrogels (i.e., P0 and P100) and the magnetic hydrogels (i.e., P0@Fe_3_O_4_ and P100@Fe_3_O_4_).

The thermal stability of the P0, P100, P0@Fe_3_O_4_, and P0@Fe_3_O_4_ hydrogels was analyzed using TGA, and the thermograms are shown in Figure 5. The corresponding thermal stability parameters are listed in Table 1. In Figure 5, the weight loss might be attributed to H_2_O elimination from the hydrogel at a temperature of approximately 230 °C. The weight loss between 230 and 570 °C was mainly due to the decomposition of poly(DMEMAB-co-NIPAm)’s main chain molecules in the hydrogel. The 50% weight loss of the hydrogel P100, P100@Fe_3_O_4_, P0, and P0@Fe_3_O_4_ were observed at 272.7, 350.1, 338.1, and 384.1 °C, respectively. The data confirmed that the magnetic hydrogels (i.e., P100@Fe_3_O_4_ and P0@Fe_3_O_4_) were more stable than the non-magnetic hydrogels (i.e., P100 and P0). This might be attributed to the incorporation of Fe_3_O_4_ into the hydrogel network.

**Figure 6:**
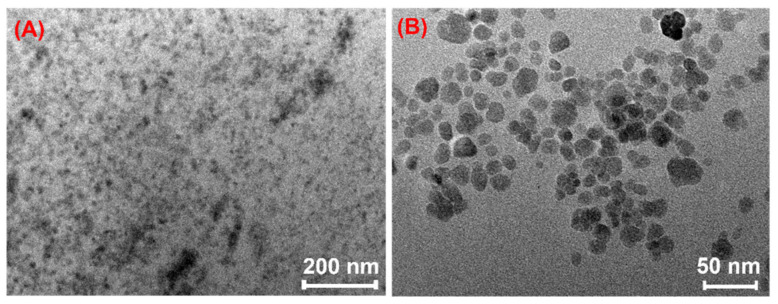
TEM images of P100@Fe_3_O_4_ at different scales: (**A**) Scale bar = 200 nm; (**B**) Scale bar = 50 nm.

The TEM images of the magnetic hydrogel P100@Fe_3_O_4_ particles are shown in Figure 6. They reveal the presence of Fe_3_O_4_ particles in this hydrogel, which were less than 10–20 nm in size. It was further proved that the magnetic hydrogel P100@Fe_3_O_4_ was successfully prepared.

### 3.3. Dye Removal Experiments

The dye removal efficiencies of P0@Fe_3_O_4_, and P100@Fe_3_O_4_ adsorbents were analyzed. They were investigated for removing BPB and RDM dyes from aqueous solutions to study the adsorption parameters. P0 and P100 were used as control samples to compare the effect of the magnetic properties on removal efficiencies. Furthermore, the adsorption time and adsorbents dosage on the BPB and RDM removal from aqueous solutions was analyzed. Batch adsorption studies were carried out to investigate the optimum adsorbent concentrations for BPB dye removal. In this study, different concentrations of the adsorbents were prepared by adding different amounts (0–50 mg) into 20 mL of BPB dye solution (0.5 mM, 350 mg/L) or RDM dye solution (0.01 mM, 5 mg/L) and stirred for 120 min. A magnet was used to separate the P0@Fe_3_O_4_ and P100@Fe_3_O_4_ adsorbents from the solution, while the P0 and P100 adsorbents were separated by centrifugation.

**Figure 7:**
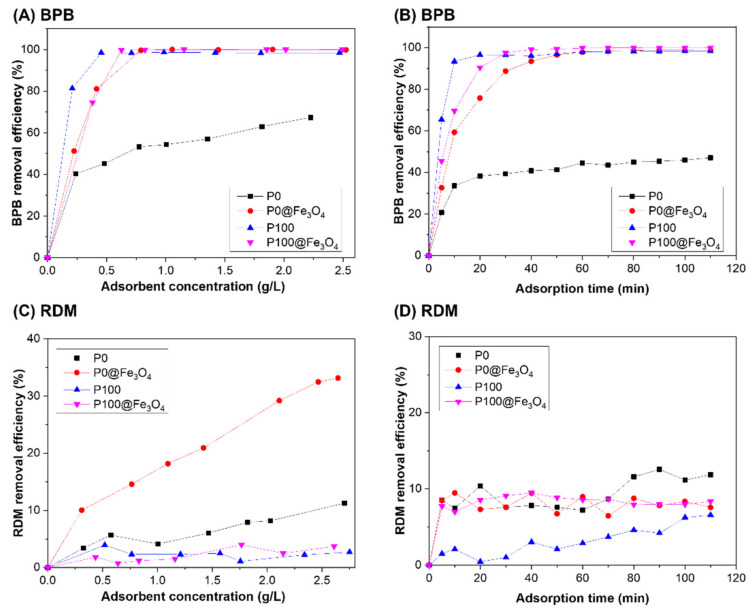
Graphs showing the removal efficiency of (**A**) BPB dye (0.5 mM, 350 mg/L) and (**C**) RDM dye (0.01 mM, 5 mg/L) as a function of adsorbent concentration for the hydrogels after 2 h of contact time. Graphs showing the removal efficiency of (**B**) BPB dye and (**D**) RDM dye as a function of adsorption time for the hydrogels. The adsorbent concentration was 0.42 g/L for the BPB solution (0.5 mM, 350 mg/L), and the adsorbent concentration was 0.62 g/L for the RDM solution (0.01 mM, 5 mg/L). All experimental data were measured at 25 °C.

**Figure 8:**
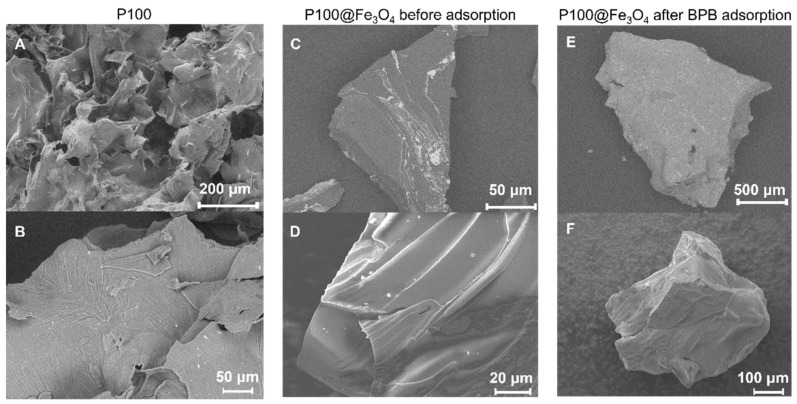
SEM images of (**A**,**B**) P100, (**C**,**D**) P100@Fe_3_O_4_ before adsorption, and (**E**,**F**) P100@Fe_3_O_4_ after BPB dye adsorption at different scales.

**Figure 9:**
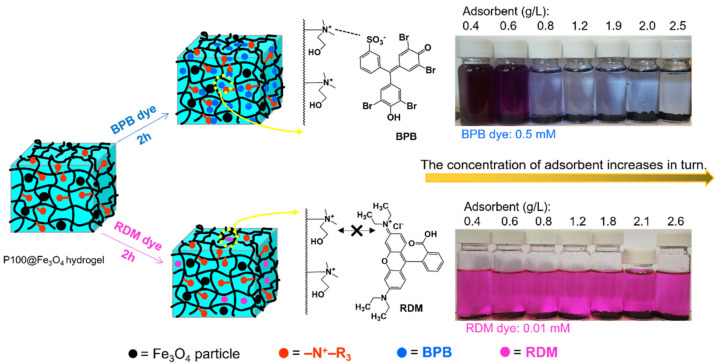
Schematic illustrations for BPB and RDM removal by P100@Fe_3_O_4_ hydrogel adsorbent.

Figure 7A shows the removal efficiencies of BPB dye from aqueous solution by the different concentrations of the adsorbents. It was found that increasing the adsorbent concentration increased the BPB removal efficiency. The BPB removal efficiency reached 98.5% at optimum concentrations of 0.455, 0.625, and 0.79 g/L of P100, P100@Fe_3_O_4_, and P0@Fe_3_O_4_, respectively. It was found that P0@Fe_3_O_4_ hydrogel had a very high removal efficiency at a concentration of 0.79 g/L, whereas P0 hydrogel could only achieve 53.3% removal efficiency at a similar concentration. This might be attributed to the increase in N^+^ positive charge density, which could significantly increase the ionic interactions with BPB dye molecules [38]. In addition, the incorporation of Fe_3_O_4_ particles might contribute to the increase in the adsorption active sites, which was conducive to the adsorption of dye molecules [39]. Another factor to consider was the molecular structure of the P100. As indicated by the SEM image of P100 (Figure 8A), P100 hydrogel had a 3D-layered structure and large surface area, which was helpful for fast adsorption of dye molecules. Based on the above reasons, the order of the performance of the four hydrogels in removing BPB dye was approximated to be P100 > P100@Fe_3_O_4_ > P0@Fe_3_O_4_ > P0. The schematic illustration for BPB removal by the magnetic hydrogel P100@Fe_3_O_4_ is shown in Figure 9.

The effect of chemical structures of the adsorbents on their dye removal efficiency and the BPB dye adsorption mechanism were also explored in the kinetics studies of BPB dye removal experiments (Figure 7B). The results showed that the removal efficiency of BPB dye increased with adsorption time, and the adsorption equilibrium was reached in approximately 30 min. The adsorption rate was very fast in the first 10 min, and the removal efficiency of BPB dye by P100 was over 90% in 20 min. The adsorption equilibrium was almost reached in 30 min, and the removal efficiency of BPB dye by P100 was 94%.

In contrast, the adsorption of RDM by these adsorbents was much less than BPB. At the maximum concentrations of P0, P0@Fe_3_O_4_, P100, and P100@Fe_3_O_4_ used in this experiment, the removal efficiency of RDM was only 11.3%, 33.2%, 2.7%, and 3.7%, respectively, as shown in Figure 7C. The removal efficiency of RDM by P0, P0@Fe_3_O_4_, P100, and P100@Fe_3_O_4_ with the adsorbent concentration of 0.62 g/L and RDM initial concentration of 5 mg/L was only 11.8%, 7.6%, 6.6%, and 8.4%, respectively (Figure 7D). The fundamental reason might be that RDM dye in aqueous solutions had positive charges and repelled each other with cationic hydrogel P100 and P100@Fe_3_O_4_, which was not conducive to dye adsorption. Moreover, the P0 hydrogel without cationic charge showed much better adsorption of RDM than P100, which further proved this hypothesis. The schematic illustration for RDM removal by magnetic hydrogel P100@Fe_3_O_4_ is shown in Figure 9.

The SEM image of the freeze-dried P100 particle in Figure 8A,B showed that the laminar structure was very obvious, and an irregular pore structure was present. This 3D porous structure could significantly increase the surface area of the hydrogel, which was conducive to accelerating dye adsorption. Figure 8C,D are the SEM images of the magnetic hydrogel P100@Fe_3_O_4_ particles before dye adsorption. The surface of the oven-dried P100@Fe_3_O_4_ particles was smooth and relatively nonporous without the 3D-layered structure of P100 hydrogel. The primary reason might be the formation of intermolecular hydrogen bonds among the –OH and CONH groups in hydrogel, inducing rapid shrinkage during in situ precipitation or the drying process. The SEM images of the oven-dried P100@Fe_3_O_4_ particles after BPB dye adsorption are shown in Figure 8E,F. Compared to P100@Fe_3_O_4_ before adsorption, its structure had no obvious change and it still had a smooth surface.

**Figure 10:**
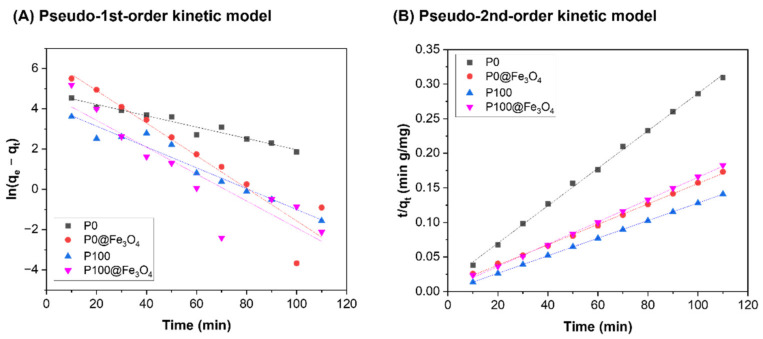
Linear fittings to the (**A**) pseudo-1st-order kinetic model and (**B**) pseudo-2nd-order kinetic model for adsorption of BPB by the hydrogels.

The kinetics data of the hydrogels were fitted into the pseudo-1st-order and pseudo-2nd-order models (Figure 10). The rate constants, *k*_1_ and *k*_2_, and the *q_e_* values are summarized in Table 2. The calculated results showed that the correlation coefficients (*R*^2^) of the two models were quite different. The adsorption kinetics data of these adsorbents fitted better into the pseudo-2nd-order kinetic model (*R*^2^ > 0.99), and the experimental *q_e_* value and calculated *q_e_* value from the pseudo-2nd-order kinetic model were also quite close. This indicates that BPB dye adsorption includes chemical adsorption and physical adsorption [21].

### 3.4. Recycling Studies

**Figure 11:**
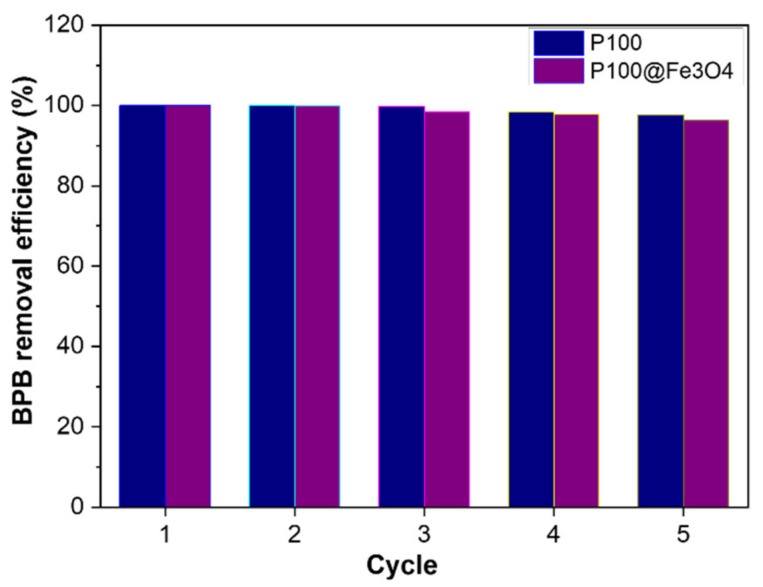
The BPB removal efficiency by P100 and P100@Fe_3_O_4_ hydrogels for five consecutive adsorption/desorption cycles at 25 °C.

The recycling studies of the hydrogels were conducted using 1 g/L of the adsorbents and 0.5 mM BPB solution at 25 °C. After adsorption of BPB, the desorption of BPB from the magnetic hydrogel P100@Fe_3_O_4_ and the non-magnetic hydrogel P100 was carried out in weakly acidic solutions (pH = 6) to regenerate the adsorbents before they were used again in the next cycles. The washing time needed to desorb BPB dye from the magnetic hydrogel P100@Fe_3_O_4_ and the non-magnetic hydrogel P100 was 2 and 5 min, respectively. The BPB removal efficiency for five consecutive cycles of adsorption/desorption of the magnetic hydrogel P100@Fe_3_O_4_ and the non-magnetic hydrogel P100 are shown in Figure 11. These data show that these two adsorbents can be recycled and used for five times without a significant decrease in their BPB removal efficiency, which was 97.7% for P100 and 96.4% for P100@Fe_3_O_4_, respectively.

## 4. Conclusions

In this study, the magnetic hydrogel, poly(DMAEAB-co-NIPAM)@Fe_3_O_4_, was successfully prepared and showed good selectivity for anionic dyes as an effective adsorbent for the removal of dye from aqueous solutions. The hydrogel poly(DMAEAB-co-NIPAM) was firstly synthesized, followed by incorporation of Fe_3_O_4_ into the hydrogel. The successful synthesized magnetic hydrogel was characterized by FTIR, XRD, TGA, and SEM. Subsequently, the adsorption behaviors of the non-magnetic and magnetic hydrogels were evaluated using BPB and RDM dyes. Various adsorption parameters, including adsorbent amount and adsorption time, were analyzed. The magnetic hydrogels showed an excellent BPB removal efficiency compared to the removal of RDM. The optimum adsorbent concentration for 0.5 mM BPB solution was approximately 0.5 g/L, and the removal efficiency was more than 99%. The kinetics data of BPB removal fitted well into the pseudo-2nd-order model, indicating that BPB dye adsorption involves chemical adsorption and physical adsorption. Furthermore, the reusability of the magnetic hydrogel for BPB removal was examined for up to five cycles and it could be reused without losing its high removal efficiency. The magnetic hydrogel, poly(DMAEAB-co-NIPAM)@Fe_3_O_4_, with high removal efficiency and good selectivity and reusability shows great potential for removal of anionic dyes in wastewater treatment.

## Figures and Tables

**Table 1:** Synthesis parameters and thermal stability properties of the hydrogels.

Hydrogel	Feed Ratios for Copolymer Hydrogels				Thermal Stability Parameters ^1^	
	DMAEMA	NIPAm	2-Bromoethanol	MBA	T_10% weight loss_ (°C)	T_50% weight loss_ (°C)
P0	1	1	-	0.03	202.6	338.1
P0@Fe_3_O_4_	1	1	-	0.03	233.1	384.1
P100	1	1	1	0.03	139.5	272.7
P100@Fe_3_O_4_	1	1	1	0.03	221.4	350.1

^1^ Data obtained from TGA analysis.

**Table 2:** Fitting parameters of adsorption kinetics of non-magnetic and magnetic hydrogels.

Adsorbents	Pseudo-1st-Order			Pseudo-2nd-Order			Experimental Values
	*q_e,cal_* (mg/g)	*k*_1_ (L/mg)	R^2^	*q_e,cal_* (mg/g)	*k*_2_ (g/mg min)	*R* ^2^	*q_e,exp_* (mg/g)
P0	119.18	0.0281	0.9540	369.00	4.66 × 10^−4^	0.9986	355.91
P100	65.04	0.0517	0.9475	787.40	1.85 × 10^−3^	1.0000	781.33
P0@Fe_3_O_4_	680.66	0.0809	0.9062	675.68	2.54 × 10^−4^	0.9988	635.72
P100@Fe_3_O_4_	117.06	0.0667	0.8197	621.12	6.43 × 10^−4^	0.9993	602.75

## Data Availability

Data are available from the authors. Samples of the compounds are available from the authors.
